# A Case of Acute Migraine Without Aura Treated With a New FDA-Approved Medication Ubrogepant

**DOI:** 10.7759/cureus.66721

**Published:** 2024-08-12

**Authors:** Maria A Shamsi, Mohammad A Khalid

**Affiliations:** 1 Medicine, Royal College of Surgeons in Ireland - Medical University of Bahrain (RCSI-MUB), Busaiteen, BHR; 2 Internal Medicine, George Washington University School of Medicine and Health Sciences, Silver Spring, USA

**Keywords:** outpatient management, headache duration, ubrelvy, calcitonin gene-related peptide antagonists, migraine relief, migraine disability index scale (midas), pain severity, migraine treatment, migraine without aura, migraine disorder

## Abstract

Ubrogepant is the first medication that blocks calcitonin gene-related peptide (CGRP), a protein released during a migraine attack, from binding to its receptors. Ubrogepant has shown positive safety, efficacy, and tolerability results for the treatment of acute migraine in phase 3 randomized trials. At this time, there are very few case reports on ubrogepant. Herein, we describe a complex patient with treatment-resistant migraine who showed substantial improvement in migraine severity, duration, and overall disability after using ubrogepant.

A 46-year-old woman with a 25-year history of migraine presented with an improvement in headache severity, duration, and disability after using a new FDA-approved medication, ubrogepant, for less than five months. Before commencing ubrogepant,her headache duration hours ranged from 36 to 60 hours, headache severity was rated 7.5/10, and mean headache days ranged from 10 to 12 days. After taking ubrogepant, her headache duration was less than 12 hours, headache severity was 3/10, and mean headache days was five.

Previously, she had been prescribed a combination of first-line medications with little improvement in headache severity. Her Migraine Disability Assessment (MIDAS) score showed moderate disability resulting in missed work and lower quality of life. To date, there have been no case reports showing the efficacy of the new FDA-approved medication, ubrogepant, showing a greater than 50% decrease in headache duration.

## Introduction

Migraine is one of the most common neurological diseases, with a global prevalence estimated to be 1.1 billion cases in the year 2019 alone [1-3]. Migraine without aura, also called “common migraine,” is listed in the Appendix of the International Classification of Headache, Third Edition (2018) [4]. Based on the classification, migraine without aura is characterized by recurrent episodic head pain that is often throbbing in nature, unilateral, and ranges from moderate to severe intensity. The migraine attacks are usually associated with nausea, vomiting, or sensitivity to light, sound, or movement. When untreated, these attacks typically last four to 72 hours.

Management

Polypharmacy is a common practice for treatment-resistant migraines, especially when symptoms are debilitating [2]. First-line medications for migraines include acetaminophen, non-steroidal anti-inflammatory drugs (NSAIDs), as well as triptans, depending on migraine's severity. Second-line medications include antiemetics and dihydroergotamine [4]. Anti-depressants and anti-epileptics may also be used depending on patient co-morbidity.

Management can be divided into non-pharmacological and pharmacological therapies [5]. It can also be simplified into abortive and preventative treatments [6].

Non-pharmacological therapies include but are not limited to trigger avoidance, sleep modification, dietary modification, maintaining a regular exercise regime depending on co-morbidity, weight reduction, management of anxiety and stress, addressing hormonal factors, and, lastly, improving patient knowledge [5].

Preventative (prophylactic) treatment

The goal of preventive therapy is to reduce the frequency, intensity, and duration of migraine episodes. Additionally, preventive therapy can enhance the effectiveness of acute migraine treatment and enhance overall quality of life.

The most commonly used medications to prevent migraines are beta-blockers (propranolol and timolol) that relax blood vessels, anti-seizure drugs (divalproex sodium and topiramate) that block nerve pain, and amitriptyline, an antidepressant that increases levels of certain brain chemicals to help alleviate migraine pain [6].

There are also newer treatments called calcitonin gene-related peptide (CGRP) inhibitors that block a brain chemical responsible for transmitting and intensifying pain signals. These can be taken as daily pills (rimegepant - also abortive medication) or monthly injections (erenumab, fremanezumab, and galcanezumab) [6].

It is important to note that prophylactic treatment does not provide a cure, and most patients will still require abortive medications for acute migraines.

Abortive (acute) treatment

The objective of acute therapy, also known as abortive therapy, is to halt a migraine as soon as it begins. Acute medications are designed to stop a migraine either at the onset or when symptoms are already present [7].

Abortive treatments demonstrate greater efficacy when administered early in the headache's progression; a substantial single dose is generally more effective than multiple smaller doses. In certain patients, oral medications may be less effective due to inadequate absorption resulting from migraine-related gastric stasis and vomiting. Most importantly, acute medications can be administered through self-injection, oral intake, skin patch, or nasal spray. These methods of medication delivery are particularly beneficial for individuals experiencing nausea or vomiting as a result of their migraine, and they provide rapid relief [7].

Migraine abortive treatment includes basic analgesics like NSAIDs or acetaminophen to triptans, antiemetics, CGRP antagonists (ubrogepant), lasmiditan, and dihydroergotamine [5,7].

In summary, pharmacological therapy selection depends on patient-specific factors, including the severity and character of symptoms, comorbid conditions, and prior response to treatment [5,6].

Emergency

Sumatriptan subcutaneous injection, antiemetics-dopamine receptor blockers, prochlorperazine intravenous (IV) or intramuscular (IM), metoclopramide, chlorpromazine IV infusion, dihydroergotamine IV, and ketorolac IV or IM are used in emergency settings - severe migraine attacks with no relief from acute migraine medications [5].

New medication

Ubrogepant is an orally administered CGRP receptor antagonist (gepant) for the acute treatment of migraine with or without aura in adults and is an option for a wide range of patients who experience migraine attacks. Ubrogepant was approved by the FDA on December 23, 2019, and is the first oral medication designed to directly block CGRP, a protein released during a migraine attack, from binding to its receptors [2].

## Case presentation

Diagnosis and clinical course

We present a case of a 46-year-old Colombian-Italian woman with a 25-year history of recurrent acute migraines. Her throbbing pain is localized at the right frontal forehead and is preceded by sensitivity to smell, nausea, and fatigue. During migraine attacks, she experiences severe neck stiffness, nausea, and pain. Physical activity makes the pain worse, and it is not relieved by rest. Previously, she used to take over-the-counter (OTC) medication Tylenol 500 mg during a migraine attack to reduce the pain and duration, but it failed to resolve the headache fully. The duration of the attack varied from two to three days. On average, she rated the headache pain severity a 7.5/10 during a migraine attack. Her Migraine Disability Assessment (MIDAS) score, a questionnaire used by doctors to assess the impact of migraines in daily life, showed moderate disability resulting in missed work and lower quality of life[8].

The patient reports three potential migraine triggers. She identified alcohol as a trigger during her 20s and discontinued all alcohol consumption from then on. The ingestion of the artificial sweetener Truvia also appears to be a trigger. Further, she reports unrestful sleep the day before a migraine attack, suggesting this may also be a trigger. Her menstruation cycle is regular and only rarely does she get migraines in the immediate days before her menses.

She has a positive family history of migraines including her maternal grandfather, mother, and aunt. Her medical co-morbidities include depression, attention-deficit/hyperactivity disorder (ADHD), and hypothyroidism. She takes Lexapro, Strattera, and levothyroxine as instructed for these conditions, and they are considered to be well-controlled.

The patient used multiple migraine treatment regimens with little to no improvement until she was prescribed Ubrelvy in October 2023 (Table 1). Her first medication used to treat migraine attacks was Migrinon tablets prescribed by a doctor in Colombia. She added a second medication amitriptyline 25 mg once a day since her migraine severity was not controlled. However, amitriptyline only improved her depression co-morbidity (Table 1). Therefore, her mainstay treatment for migraine was diclofenac combined with Migrinon and amitriptyline. She had multiple hospitalizations in her 20s-30s of severe migraine attacks - with one attack lasting one week. She used the NSAID diclofenac injection during her 20s and 30s when she had severe migraines.

**Table 1:** Medication history of the patient. IM: intramuscular.

Medication	Mechanism of action	Time period
Migrinon (medication brand found in Columbia), dipyrone (metamizole) 300 mg, isometheptene 30 mg, caffeine 30 mg oral once a day	Inhibition of a central cyclooxygenase-3 and activation of the opioidergic system and cannabinoid system - a sympathomimetic amine, which constricts cerebral blood vessels and reduces pulsation in cerebral arteries	First medication during the time of diagnosis. Stopped medication completely after commencing Ubrelvy^®^ inOctober 2023
Amitriptyline 25 mg oral at bedtime	Unclear mechanism but it is thought to inhibit norepinephrine and serotonin uptake, block sodium channels, enhance GABA-mediated inhibition, potentiate endogenous opioids, and intensify descending inhibition on nociceptive pathways	Stopped medication 2 years ago and switched to a selective serotonin reuptake inhibitor (Lexapro) for depression/anxiety co-morbidity
Diclofenac 50 mg oral once a day	Non-steroidal anti-inflammatory medication. It inhibits the cyclooxygenase (COX), which catalyzes the production of prostaglandins responsible for pain and inflammation	The second medication used after no improvement with Migrinon. Stopped medication after being prescribed Ubrelvy^®^ in October 2023
Naproxen 1 g/2 ml IM	Same as above but injection formulation	Used during emergency settings
Diclofenac injection 25 mg/ml IM	Same as above but injection formulation	Used during emergency settings
Ubrogepant100 mg once a day	Calcitonin gene-related peptide antagonist	Began in October 2023 with a lower dose of 50 mg, then increased to 100 mg once a day in December 2023

Patient self-reported feedback

**Figure 1:**
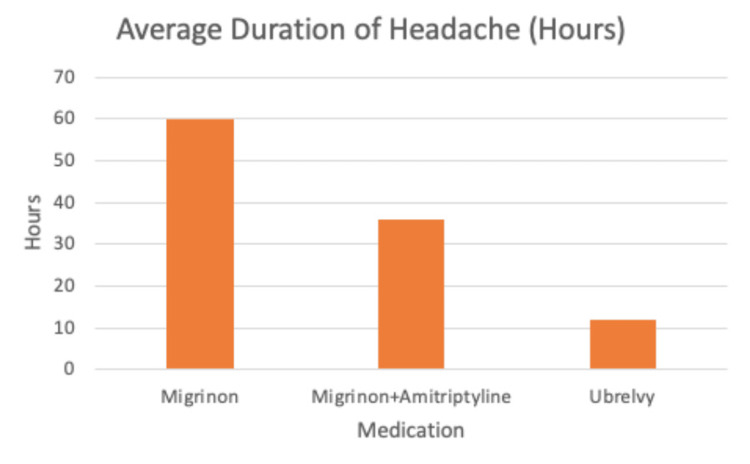
The average duration of headache in hours while on Migrinon, Migrinon and amitriptyline combination, and ubrogepant. The patient's average headache duration decreased by 30% after taking ubrogepant (36 hours to 12 hours). Note: Ubrogepant is sold under the brand name Ubrelvy.

**Figure 2:**
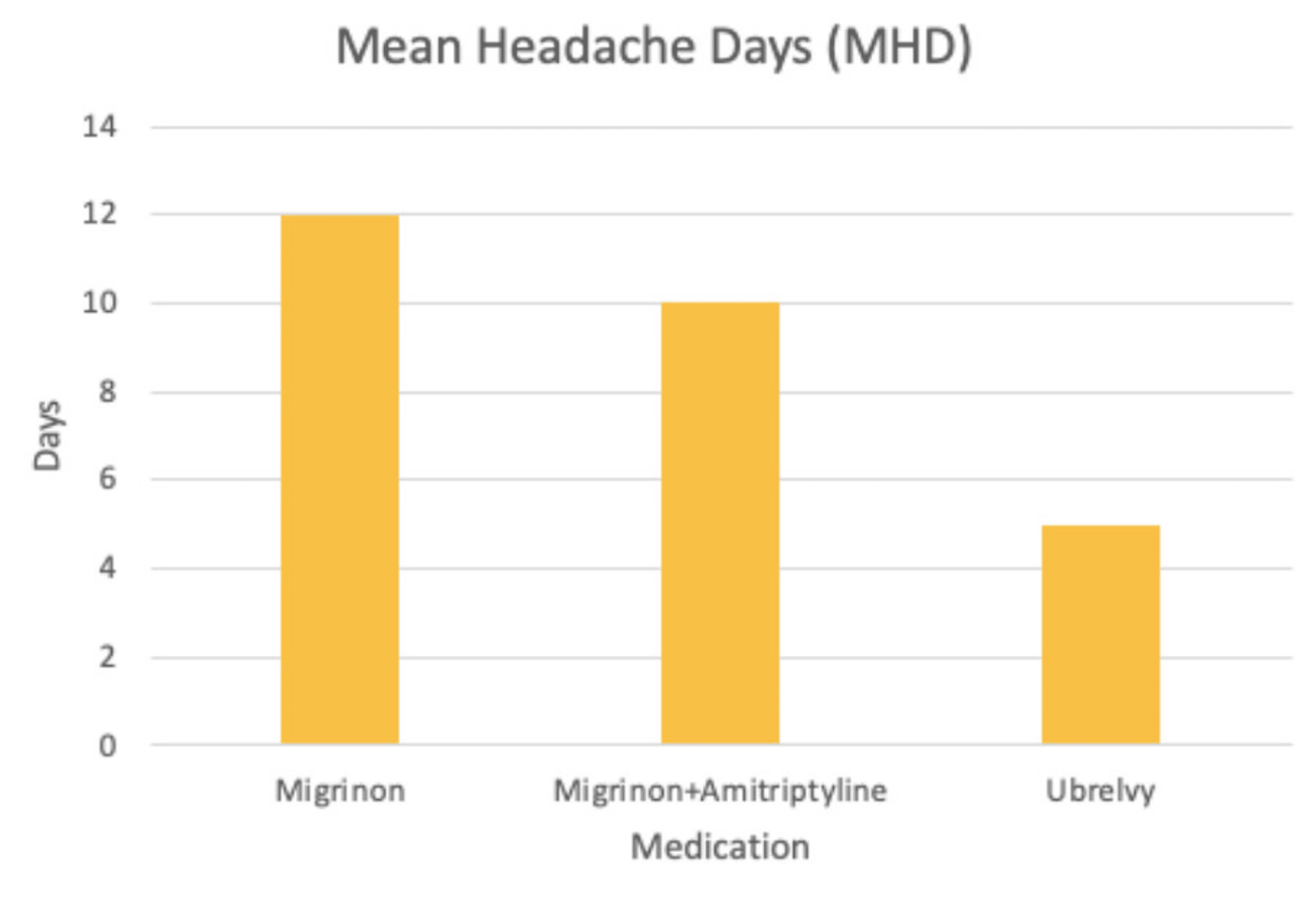
Mean headache days over a 30-day period (one month) while on Migrinon, Migrinon and amitriptyline combination, and ubrogepant. The patient's mean headache days were decreased by 50% after taking ubrogepant (12/30 days to 5/30 days). Note: Ubrogepant is sold under the brand name Ubrelvy.

**Figure 3:**
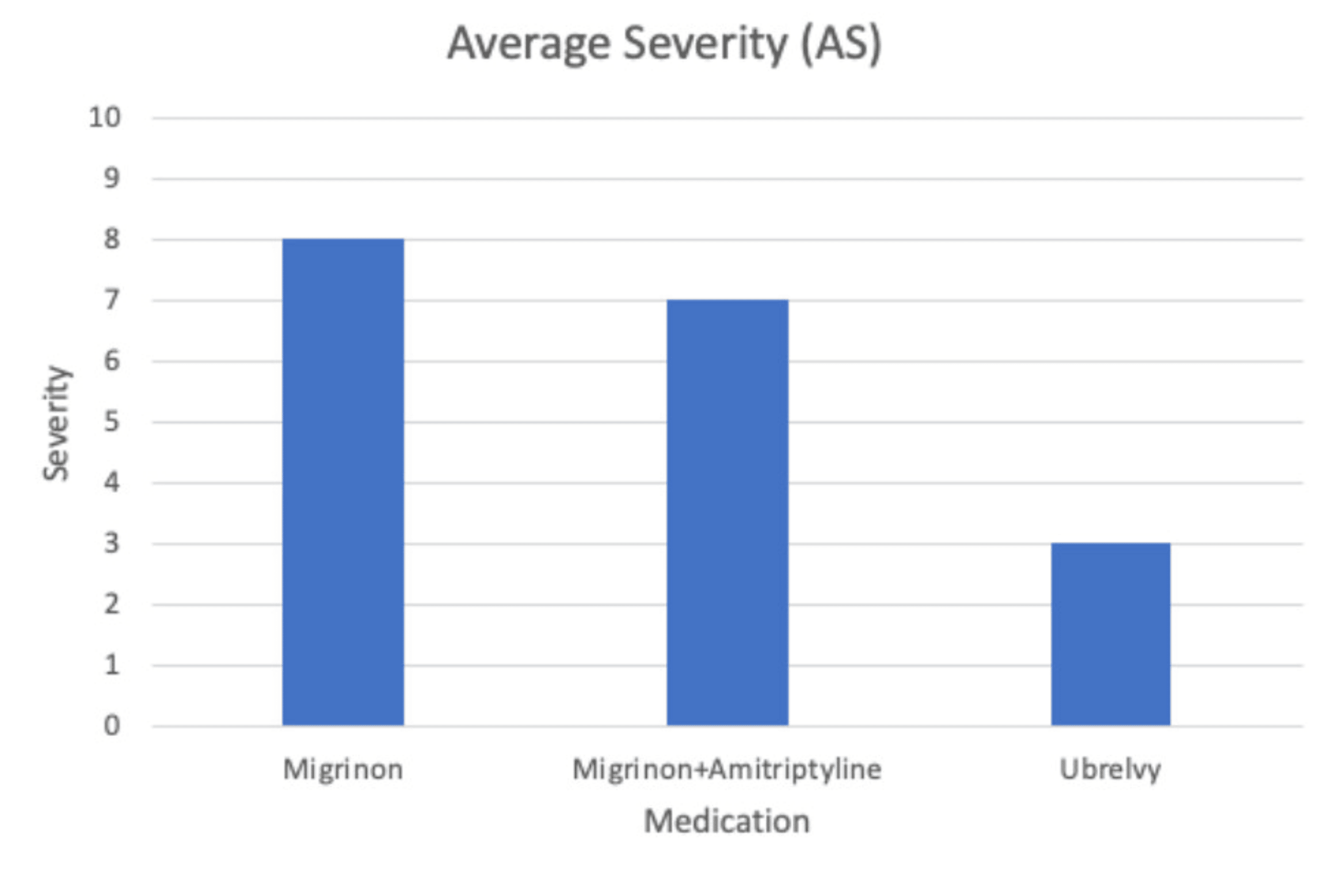
Average headache severity while on Migrinon, Migrinon and amitriptyline combination, and ubrogepant. The patient's headache severity decreased by 50% after taking ubrogepant (8/10 to 3/10 pain scale). Headache severity was estimated using the Migraine Disability Assessment (MIDAS) questionnaire. The patient kept an active headache diary. Note: Ubrogepant is sold under the brand name Ubrelvy.

The patient reported less duration of headache attacks and mean headache days (Figures 1, 2). She also noted that ubrogepant helps with her nausea symptoms more quickly and effectively than the other medications. The patient has also improved her sleep posture hygiene and purchased a pillow to help her neck stiffness and headaches. Lastly, her MIDAS score decreased, reinforcing improvement in her migraines with ubrogepant (Figure 3).

## Discussion

We present a patient with long-term acute migraine who kept track of her migraines in detail with headache days, duration, and severity over the years, which gave us objective evidence to compare the efficacy of migraine preventative treatments. She had been prescribed three different combinations of medications over the past years for her migraines (Migrinon, amitriptyline, and diclofenac), but these medications failed to provide relief until she used the new FDA-approved medication ubrogepant.

There is an increasing amount of awareness in the literature supporting CGRP in migraine pathogenesis and treatments targeting CGRP receptors.

Mechanism of action of CGRP in migraine attack

Although the neural mechanism of migraine pathophysiology is quite complex, many clinical studies have identified CGRP as a key molecular player. It is proposed that the neuropeptide - CGRP - is released when the trigeminal ganglion is stimulated during migraine attacks [9]. This leads to meningeal vasodilation and dural mast cell degranulation, which contribute to neurogenic inflammation. The blood pulsing through dilated cerebral vessels and inflamed arteries causes the pulsatile, pounding headache known as migraine. As the migraine progresses, the thalamus - the “pain relay center” of the brain - becomes desensitized. During central sensitization, which is the worst part of migraines, a faint touch may be perceived as painful. For example, some migraine sufferers will complain their head is sensitive to touch or pain is felt when they wear glasses or lie on one side of their pillow. This is called allodynia, a clinical sign of central sensitization [9].

Mechanism of action of ubrogepant in migraine alleviation

Ubrogepant intervenes with the "trigeminovascular system" as mentioned above. In simplified terms, the "trigeminovascular system" can be called the "CGRP cascade pathway," which involves trigeminal nerve fibers releasing CGRP, leading to cerebral blood vessel dilation and pain signal transmission to the brain. This process also releases other pro-inflammatory agents (mast cells, 5-hydroxytryptamine, cyclooxygenase-2), which exacerbate migraine's inflammation and discomfort [10].

As a CGRP receptor antagonist, ubrogepant blocks this receptor, preventing CGRP from binding and exerting its effects [9]. Therefore, there is a reduction in blood vessel dilation, thalamus desensitization, and the release of inflammatory agents, resulting in alleviation and eventually termination of migraine symptoms.

Comparison of oral CGRP antagonists with placebo, triptans, and 5-hydroxytryptamine 1F receptor agonists

To our knowledge, there are no specific studies comparing the different types of oral CGRP antagonist medications. However, in terms of pain freedom at two hours post-dose and pain relief at two hours post-dose, a recent meta-analysis concluded that CGRP antagonists were significantly more effective than placebo [11]. Also, the meta-analysis concluded that the CGRP antagonists were less effective than the triptans with respect to outcomes such as pain freedom at two hours post-dose and pain relief at two hours post-dose [11]. Another systematic review concluded triptans, 5-hydroxytryptamine 1F receptor agonists, and CGRP antagonists decrease migraine pain at the two-hour mark when compared to the placebo. Most triptans showed a reduction in pain when compared to 5-hydroxytryptamine 1F receptor agonists and CGRP antagonists. However, 5-hydroxytryptamine 1F receptor agonists exhibited the highest likelihood of adverse events among all treatments, while selective triptans showed a higher risk of adverse events compared to CGRP antagonists [12].

Ubrelvy’s safety profile

The safety profile of Ubrelvy® has been evaluated in several clinical trials, with no treatment-related death, indicating a generally favorable safety profile. The most common adverse reactions of Ubrelvy® in the ACHIEVE II randomized clinical trial were nausea (4% vs. 2% placebo) and somnolence (3% vs. 1% placebo) [13].

Clinical trials on Ubrelvy’s efficacy and our patient

In the ACHIEVE II randomized clinical trial, 20.8% of migraine patients achieved freedom from pain at two hours post-dose with either 25 mg or 50 mg of ubrogepant, compared to just 12.6% with placebo (p < 0.001). Furthermore, in the ACHIEVE I trial, the return to normal function at two hours was significantly higher in ubrogepant groups (45% on 100 mg, 40.9% on 50 mg) than with placebo (29.5%; p < 0.001 for both). Our patient’s headache duration decreased from 36 hours to 12 hours post-dose (Figure 1), emphasizing ubrogepant as an effective migraine treatment [2,14].

This case clearly shows us the positive effect of Ubrelvy® on migraine severity, duration, and overall disability. However, there are several limitations above and beyond those inherent in singular case reports such as this that should be considered when interpreting the treatment outcome of this patient, in addition to being a single case report. Firstly, the ACHIEVE clinical trial included adult participants with migraine with or without aura experiencing two to eight migraine attacks per month. Our patient started with 12 headaches per month (Figure 2). Secondly, the clinical outcomes for the trials were different than our case report, but overall, both the trials and our case confirmed a decrease in pain and severity of migraines and increased efficacy of ubrogepant. Lastly, lifestyle modifications such as improving sleep posture and purchasing a new pillow might have improved the patient’s migraine, rather than the new medication.

Nevertheless, this case report still demonstrates the importance and potential of Ubrelvy® as a successful new FDA-approved medication in managing long-term migraine sufferers who have found little relief from other pharmacological treatments.

## Conclusions

We reported a case of a patient with a 25-year history of migraines who utilized ubrogepant for a duration of less than five months. Following the administration of this newly FDA-approved medication, the patient experienced a reduction in headache severity, duration, and associated disability.

In conclusion, this case represents the importance of evaluation of the duration and frequency of the headaches, in addition to the severity, especially for patients having minimum to little relief with attempted treatments. Having a detailed headache diary could help healthcare providers find the optimal treatment for migraine individuals. It is also important for healthcare providers to incorporate new medications such as CGRP receptor antagonists for the treatment of migraines, with close monitoring of side effects, to improve patients’ quality of life.
